# Evaluation of short message service and peer navigation to improve engagement in HIV care in South Africa: study protocol for a three-arm cluster randomized controlled trial

**DOI:** 10.1186/s13063-016-1190-y

**Published:** 2016-02-06

**Authors:** Sheri A. Lippman, Starley B. Shade, Jeri Sumitani, Julia DeKadt, Jennifer M. Gilvydis, Mary Jane Ratlhagana, Jessica Grignon, John Tumbo, Hailey Gilmore, Emily Agnew, Parya Saberi, Scott Barnhart, Wayne T. Steward

**Affiliations:** Center for AIDS Prevention Studies, Department of Medicine, University of California, San Francisco, UCSF Box 0886, 550 16th Street, 3rd Floor, San Francisco, CA 94143 USA; International Training and Education Center for Health South Africa, University of Washington, 232 Bronkhorst Street, Pretoria, Republic of South Africa; Department of Global Health, University of Washington, Seattle, USA; Department of Family Medicine and Primary Health Care, University of Limpopo - Medical University of Southern Africa campus, Pretoria, South Africa; Departments of Medicine and Global Health, University of Washington, Seattle, WA USA

**Keywords:** Cluster randomized trial, South Africa, HIV, Adherence, Engagement in care, Retention, SMS, Peer navigation

## Abstract

**Background:**

In countries with a high burden of HIV, such as South Africa, where the epidemic remains the world’s largest, improving early uptake of and consistent adherence to antiretroviral therapy could bring substantial HIV prevention gains. However, patients are not linked to or retained in care at rates needed to curtail the epidemic. Two strategies that have demonstrated a potential to stem losses along the HIV care cascade in the sub-Saharan African context are use of text messaging or short message service (SMS) and peer-navigation services.

**Methods/Design:**

We designed a cluster randomized trial to assess the efficacy of an SMS intervention and a peer-navigation intervention to improve retention in care and treatment, timely linkage to care and treatment, medication adherence, and prevention behaviors in South Africa. Eighteen primary and community healthcare clinics in Rustenburg and Moses Kotane Sub-districts in the North West Province were randomized to one of three conditions: SMS intervention (n = 7), peer navigation intervention (n = 7), or standard of care (n = 4). Approximately 42 participants are being recruited at each clinic, which will result in a target of 750 participants. Eligible participants include patients accessing HIV testing or care in a study clinic, recently diagnosed with HIV, aged 18 years or older, and with access to a cellular telephone where they are willing to receive automated SMS with HIV-related messaging. Data collection includes extraction of visit information from clinical files and participant surveys at baseline, 6 months, and 12 months. Intent-to-treat (ITT) analysis will explore differences between randomization arms and the primary outcome of patient retention in care at 12 months following enrollment. We will also explore secondary outcomes including participants’ a) timely linkage to care (within 3 months of HIV diagnosis), b) adherence to treatment based on self-report and clinic’s medication dispensation dates, and c) condom-use behaviors.

**Discussion:**

The findings will allow us to compare the efficacy of two complementary interventions, one that requires fewer resources to implement (SMS) and one (peer navigation) that offers more flexibility in terms of the patient barriers to care that it can address.

**Trial registration:**

NCT02417233, registered 12 December 2014.

## Background

The success of “treatment-as-prevention” trials has ushered in a new era in worldwide HIV prevention efforts [1]. Antiretroviral therapy (ART) suppresses viral replication, effectively forestalling disease progression and reducing the likelihood of viral transmission [2–6]. The landmark HPTN 052 trial among HIV sero-discordant couples found that early ART initiation (when CD4+ cell count is between 350 and 500 cells/μL) can reduce HIV transmission to uninfected partners by 96 % [2]. In countries with a high burden of disease, such as South Africa, where the epidemic remains the world’s largest with an estimated 6.4 million people living with HIV (PLHIV) [7], improving early uptake of and adherence to ART could bring substantial gains in HIV prevention. Modeling suggests that annual HIV testing with the immediate initiation of ART (at any CD4 count) in South Africa could reduce HIV incidence and mortality to less than one case per 1000 people per year by 2016 [8]. Additionally, South Africans residing in communities with greater ART coverage have a lower risk of HIV acquisition [9]. While instituting universal ART coverage will be challenging to implement in resource-constrained settings, important gains can be attained by ensuring that infected individuals are identified early in their HIV disease, enter into care rapidly, and remain in care and adherent to their regimens following the initiation of treatment. Unfortunately, in sub-Saharan Africa, some 40 % of individuals who test positive are not successfully linked to care, and only 50 % of those who are not yet ART-eligible are retained in HIV care [10], leading to a “cascade” of losses at each stage along the care continuum [11].

In South Africa, the government continues to sponsor national HIV testing campaigns and has expanded ART eligibility, first to those with a CD4+ cell count of 350 cells/μL or less and recently to those with a CD4+ cell count of 500 cells/μL or less [12, 13]. Although treatment expansion is critical, current losses along the HIV care cascade are impeding HIV prevention gains. National data from 2012 indicated that 65 % of the population had been tested for HIV; however, only 37.8 % of HIV-positive men and 55 % of HIV positive women were aware of their HIV status [7]. Additionally, once diagnosed, many do not initiate care or treatment. A study in Cape Town found that only 62.5 % of recently diagnosed patients received a CD4+ test result within 6 months; 66.7 % of those that were eligible for ART initiated treatment; and only 46.3 % of those not ART eligible returned for a subsequent CD4+ test [14]. In a study in rural Kwa-Zulu Natal, only 44.9 % of the HIV-positive patients not yet ART eligible returned for subsequent CD4+ tests within 13 months [15]. To summarize, whereas South Africa has the largest antiretroviral program in the world [16], only 52 % of patients eligible for ART are estimated to currently receive treatment [17]. These failures to engage patients in care are exacerbated by further considering loss to follow-up after ART initiation, which has long-lasting implications, worsening outcomes for PLHIV, even if they return to care [18].

Interventions that link and retain patients in HIV care, promote ART adherence, and encourage risk-reduction behaviors in resource-limited settings are essential and could substantially improve survival and prevent new HIV infections [2, 19, 20]. We sought to identify strategies that do not require extensive resources and could realistically be implemented and supported by health departments in sub-Saharan Africa. Among feasible strategies that have the potential to improve care engagement outcomes, particularly retention and adherence, text messaging or short message service (SMS) and personal support have both demonstrated promise and could be economically feasible [20–22]. SMS cost 1 to 2 cents to send in bulk and are free for clients to receive. Systematic reviews and meta-analyses have demonstrated that the use of SMS for appointment reminders have improved clinic attendance [23–25]. Additionally, SMS interventions that included appointment reminders, laboratory result notifications, and medication reminders have demonstrated promise in improving ART initiation and retention in care, as well as ART adherence in African settings [26–29]. One randomized trial also demonstrated improved rates of viral suppression among those receiving weekly SMS [30].

Similarly, personal support interventions that focus on professional case management or engaging peers or family networks to provide encouragement and support to remain in care and adhere to medications have demonstrated promise [22]. A study in Kenya found that the time to treatment failure was longer (reflective of better retention and adherence to ART) for patients who participated in support groups, received pharmacy counseling, or who had home visits [31]. Research in Uganda suggests that care outcomes for those initiating ART and those still pre-ART can be improved when patients receive treatment support [32, 33]. Furthermore, recommendations from an international expert physician panel now call for systematic monitoring of linkage and retention in care, including the potential use of peer or paraprofessional patient navigators to assist patients in obtaining the care that they need [34]. Furthermore, peer navigators are less costly than professional personnel and may be more feasible to support in a resource-constrained setting.

Given the promise of these intervention options, we designed a cluster randomized trial to assess the efficacy of an SMS intervention and a peer navigation intervention to improve retention in care, timely linkage to care and treatment, ART adherence, and prevention behaviors in South African primary health clinics in the North West Province.

## Methods/Design

### Study design

**Fig. 1:**
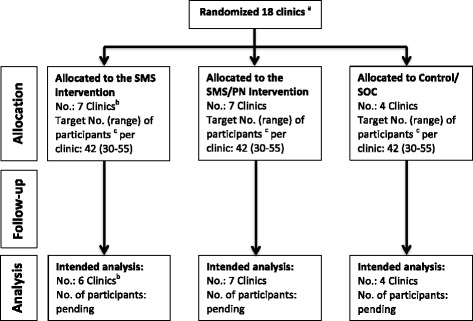
Clinic allocation and participant enrollment. Notes: ^a^ Clinics were not eligible if they had a catchment area under 6,000 inhabitants, inadequate patient load to ensure recruitment targets (40 new patients to patient registers in the first 4 months of 2014), were pilot clinic sites (n = 4). ^b^ One clinic assigned to the SMS arm was under construction and has not opened as scheduled; as such, recruitment targets have been adjusted at the remaining SMS clinics. ^c^Participants are eligible if they are 18 or older, diagnosed with HIV within the last 12 months, receiving care at a study clinic, and willing to receive communication from study staff. Abbreviations used in the figure: No. = Number; PN = Peer Navigation; SMS = Short Message Service (i.e., Text Message); SOC = Standard of Care

Using a cluster randomized trial at the level of the clinic, we will evaluate the efficacy of employing two strategies for enhancing linkage to and retention in clinical care, ART adherence, and HIV preventive behaviors. A target of 750 participants will be enrolled across 18 clinics in the Moses Kotane and Rustenburg Sub-districts, Bojanala Platinum District, South Africa (Fig. 1). Clinics have been randomized to one of three conditions: an SMS intervention arm, a peer navigation (PN) arm that also includes SMS, and a control condition. Clinics have been randomized instead of individuals to avoid contamination of intervention across individuals within clinics and to facilitate cooperation with clinic staff. Participant outcomes will be monitored using both clinical record extraction to document HIV care visits and services and a series of interviewer-administered surveys at enrollment (baseline assessment), 6 months after baseline, and 12 months after baseline.

### Study setting

The North West Province has a population of just over 3.5 million people. The province is disproportionately young, with 60 % of the population under the age of 34 years, with high rates of unemployment, poverty, and low levels of educational attainment. Over 46 % of the population is living in poverty compared to 39.9 % of the national average [35]. The province has the fourth highest HIV prevalence in the country: 13.3 % among the general population [7] and 29.7 % among women in antenatal services [36]. Within North West Province, the Bojanala Platinum District, representing 37 % of the overall provincial population, has an HIV prevalence of 35.0 % in the antenatal clinics [36], making it more heavily affected than most other districts. Care in Bojanala Platinum is provided by one provincial hospital, four district hospitals, 13 community health centers, 104 primary health clinics, and numerous mobile health points. Our study area was limited to two sub-districts, Moses Kotane and Rustenburg, and facilities included community health centers and primary health clinics only.

### Clinic selection and randomization

The 18 study clinics were selected from among 61 ART-initiating clinics within the two study sub-districts. To be eligible, a clinic could not be one of the four that had participated in a pilot test of the interventions, had to have a catchment area of 6,000 or more inhabitants, and have adequate patient load to ensure recruitment targets (defined as having 40 new individuals added to HIV patient care registers in the first 4 months of 2014). From among the 24 clinics meeting these criteria, we excluded one clinic that primarily served the mobile mining workforce (estimated at over 90 % of their patient load), and therefore was unlikely to retain patients. We also excluded one clinic that was not using patient registers, which precluded establishing eligibility; one clinic that was scheduled to close; and three clinics that were distant from study offices, complicating access and supervision.

The 18 remaining clinics were randomized to one of the three study arms, including seven to each intervention condition and four to standard of care (Fig. 1). The decision to randomize more clinics to intervention conditions was based on agreements with the local government partners at the district of health. We randomized sites using methods described by Hayes [37]. We stratified by sub-district and employed balanced (restricted) randomization based on number of total monthly clinic visits, number of HIV tests conducted in the previous four months, number of patients on ART, and the functionality of the monitoring and evaluation systems (including record-keeping and supply chain management) to ensure balance across the study arms.

### Participant eligibility and recruitment

We will be enrolling 750 participants, with a target of 42 (range = 30-55) participants at each facility. All participants must be diagnosed with HIV within 1 year preceding their enrollment. Participants must also be 18 years or older, have access to a cellular telephone where they are willing to receive automated text messages with HIV-related content, and be willing and able to give informed consent (not under the influence of illicit substances, or seemingly emotionally unstable at the time of recruitment and enrollment). Written informed consent will be obtained from all participants prior to enrollment in the study.

We aim to recruit an equivalent number of men and women and ensure that the sample has substantial representation (minimum of 30 %) of clients who do not yet quality for ART in order to assess the linkage and retention for both clients who initiate ART and those who do not. To accomplish this balance, recruitment will be completed using two strategies. The first strategy includes recruiting clients who present at the clinic for HIV testing and receive a positive diagnosis for the first time, or those who are returning to collect their initial CD4+ test results. Recruitment will be systematic: all of those presenting for an HIV test or for an initial CD4+ test result will be approached by clinic staff about their interest in participating in the study. We initially estimated that between 50 % and 60 % of those recruited during HIV testing would have an ART-qualifying CD4+ cell count at the time of recruitment [38]. Because the national guidelines have recently changed to increase qualifying CD4+ to 500 or less, it is more likely that 70 to 80 % of the individuals newly diagnosed after January 2015 will qualify for ART.

The second recruitment strategy includes identifying patients from recent entries in the HIV care registers at the clinic sites. This selection will also be systematic: we will enumerate every male and female in the HIV care registers and then systematically select men and women at pre-determined intervals. The selection interval (for example, every fourth woman listed) will differ by clinic depending on the total number of male and female adult registry entries. Because clinic registers are more complete for ART patients than for pre-ART patients (those not yet eligible for ART), we assume that the large majority of patients recruited from the registers (upwards of 90 %) will have qualified for ART. Therefore, we aim to recruit 75 % of our participants through the first strategy and 25 % of participants through the second strategy to ensure that non ART-qualifying participants are included.

### Intervention

Two intervention strategies to increase retention in care (primary outcome), as well as linkage to care, ART adherence, and prevention behaviors (secondary outcomes) will be compared to local standard of care (SOC). The two intervention strategies that participants will receive for the duration of 1 year are as follows: 1) an automated SMS and 2) a peer-navigator (PN) model that also includes some automated SMS.

**Fig. 2:**
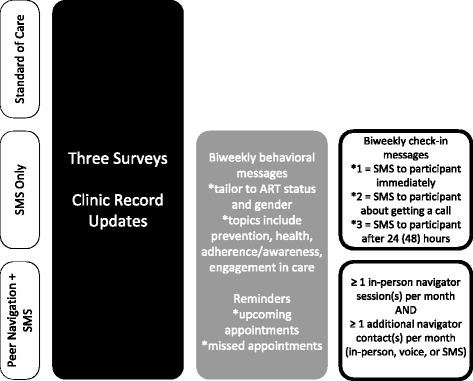
Map of the principal data collection and intervention activities across three study arms. Abbreviations used in the figure: ART = Antiretroviral Therapy; SMS = Short Message Service (i.e., Text Message)

The first strategy includes three types of messages to be sent to participants that will be delivered automatically from a central system based on their gender and clinical/ART status recorded on registration and enrollment forms and updated through clinical record extraction (Fig. 2). These three message types include the following:

1. Behavioral Messages: These messages are sent on a biweekly (every other week) basis, for a total of 26 messages during the 1-year study period. Behavioral messages are designed to address four themes: HIV prevention (some of these messages are gender-specific); engagement in care; medication adherence (for those on ART) or awareness (for those not yet eligible for treatment); and healthy living, which includes messaging concerning alcohol/drug use, social support and safe disclosure, and health/nutrition.
2. Care Reminder Messages: Participants are sent a message 3 days before any scheduled clinic visits, whether for ART refills, CD4+ or HIV viral load testing, or other routine care. Participants also receive a reminder message if they have missed a clinic visit by over 2 weeks, in line with South African guidelines [13]. Patients receive up to 6 messages (bi-weekly) following a missed appointment to encourage them to return to the clinic. Additionally, when a client has failed to present for a visit for 3 months, the study staff will alert the clinic that a client has been lost to follow-up.
3. Two-way Messages: These are automated biweekly “check-in” messages that are sent from a central system; they also record participant responses and send a system alert to the team if a follow-up call to the participant is needed. Two-way messaging serves to maintain contact with the participant, provides a means of retention in the study and in care, and also is a probe to ensure that the participant is not defaulting on care and treatment or experiencing any difficulties that need to be addressed. The participant receives a request to “reply 1 if you’re good or 2 if you have a problem and need to talk.” If the participant responds “2,” project staff will receive an alert to call the client within the first business day after receiving the notification. If the participant does not respond to the check-in message within 24 hours, the participant receives the message a second time. If the participant has not replied to either message after 48 hours, the study staff will receive an alert to call the client. When staff call for either a “2” response or a non-response, they follow a protocol for referrals and are trained not to offer advice and counseling. Responding to two-way messages is free of cost for participants.

The automated messaging system provides scheduled delivery of messages via the CommConnect online platform, part of the CommCare system developed by Dimagi, Inc. The particular behavioral messages that a participant receives are determined by gender and ART status, as captured by study staff during enrollment. The ART status classification is also linked to ongoing clinic record extraction, so that if the ART status changes, so does the message tree. Reminder messages are triggered by a “date of next appointment” field, which is updated during weekly chart extraction by study staff. If chart extraction indicates that a participant has missed a visit by over 2 weeks, a reminder is also sent. The CommConnect system sends and receives messages free of cost to participants. Even so, the study provides each participant with 50 to 60 Rand of airtime on the participant’s cell phone carrier of choice at enrollment and again at the 6-month follow-up visit to ensure that participants are comfortable using their phones for responding to study check-in messages.

The second intervention strategy is predicated on relationship building and support with fellow patients. Specifically, the strategy makes use of peer navigators, who work with participants to assess their barriers to retention in HIV care, ART adherence, and prevention of further HIV transmission, and then aids participants in developing plans and strategies for overcoming these barriers. To be eligible to serve as a peer navigator, a person must be HIV-positive, receiving care at a government-run primary healthcare facility, and have demonstrated commitment to adhering to medications and clinical monitoring. Each navigator will assist approximately 10 participants (no more than 12) during the study. Following an initial visit, navigators aim to have at least one in-person meeting and one check-in by SMS or phone with each participant each month (that is, a minimum of two contacts per month for 12 months). This standardized contact schedule is intended to serve as a minimum threshold. A navigator’s work with a participant will often involve additional contacts in the course of a month, such as offering to accompany a participant to their appointments (with participant consent), assisting a participant with disclosure of his or her HIV status to a family member or friend, or conducting additional meetings by phone or in-person to discuss emerging challenges and strategize solutions.

Participants in the peer navigator condition will also receive biweekly behavioral messages and appointment reminders, which will be sent via the CommConnect system according to the same schedule as used in the SMS-only strategy. Following the pilot of these strategies in four clinics, the study team resolved to include automated reminders and behavioral messages instead of personalized reminders and behavioral messages sent by the navigators to clients in the peer navigation arm. This decision ensures better consistency of messaging because navigators in the pilot phase struggled to personalize the sequencing of the various behavioral messages while still adhering to standardized scripts for each message. Additionally, investigators felt that automating the messages for clients in the PN arm would facilitate exploration of incremental effectiveness of the two study interventions, with one (PN) building on the other (SMS).

Peer navigators will meet weekly with their direct supervisors and biweekly with the larger study team to discuss challenges being observed among participants, and to develop potential approaches to overcome the observed challenges. In addition, the supervisory team meets weekly with study investigators to identify emerging complex challenges with particular participants or navigators, and to strategize solutions. Navigators also have access to a “clinic liaison” at each clinic site. This individual, usually one of the nurse providers, serves as a primary point of contact between the study team and clinic staff at a site. Navigators can refer their participants to the clinic liaison when, for example, they feel that a participant has a problem that requires medical attention or a referral for other medical or social services.

### Data collection procedures

Regardless of the intervention assignment, all participants, including those receiving services in the SOC clinics, are asked to complete a baseline survey assessment and provide contact information on the day that enrollment occurs. Participants will be reassessed via surveys during the 6-month and 12-month follow-ups. Both baseline and follow-up assessments will capture information about (1) access to clinics and HIV care services received in the 6 months preceding each assessment, (2) self-reported assessments of ART adherence (for participants on ART), and (3) self-reported HIV risk behaviors and risk reduction practices (condom use or partner reduction). The follow-up surveys also capture (4) self-reported receipt of the intervention components (for participants in the intervention arms) and (5) self-reported satisfaction with SMS and peer navigator services. We will also capture data related to demographics, disclosure, anticipated stigma [39], alcohol use (AUDIT-C) [40, 41], and mental health (CESD-10) [41, 42].

Data regarding clinical visits and care received will be extracted from clinical files. Record extraction will occur weekly for participants on ART, and every 2 weeks for those not on ART, who are seen less frequently than the ART patients. Study staff will capture details regarding any visit that occurred since the last extraction, including the visit date, HIV-related services received (for example, medications), and any related CD4+ or HIV viral load test results. All data collection forms and procedures have been reviewed and approved by the Committee for Human Research at the University of California, San Francisco (UCSF), the Human Subjects Division at University of Washington, and the Human Sciences Research Council (HSRC) Research Ethics Committee in South Africa. The Policy, Planning, Research, Monitoring and Evaluation Committee for the North West Provincial Department of Health also reviewed and approved the protocol.

We will also conduct microcosting of the resources needed to carry out the activities in all arms of the intervention. Costing will include the following: 1) personnel (including fringe benefits), 2) recurring supplies and services, 3) capital and equipment (for example, furniture and computers), and 4) facility space. Intervention costs will be assessed using a uniform cost data-collection protocol to quantify resources used and associated costs in each of the study arms. Data will be obtained through administrative record reviews and administrative and program staff interviews, supplemented by a staff time study in order to distinguish pre-implementation and implementation activities. Pre-implementation includes hiring, procurement, intervention development/modification and training. Implementation includes recruitment, intervention activities and supervision. Nonresearch program management costs will be allocated based on the period in which they occurred (pre-implementation versus implementation).

### Measures

Primary and secondary exposure and outcome measures are included in Table 1. Primary exposure for the intent-to-treat analysis is classified as the clinic randomization arm: SMS intervention, PN intervention, and SOC. The primary study outcome is retention in care, which is defined for patients on ART as the proportion of patients who have initiated ART who remain on treatment at 6 and 12 months and for patients who are not yet eligible as the proportion of patients who undergo a repeat CD4 test at 6 months and 12 months after the initial CD4. The secondary outcome of linkage to care is defined as a patient undergoing CD4 staging or being assessed for ART eligibility within 3 months of a positive diagnosis. We will also monitor whether the participants are linked to ART – that is, whether eligible participants receive ART within 3 months of CD4 staging. The secondary outcome of adherence is being assessed in three ways: as a report of taking 90 % of the prescribed medication, as no report or clinical record indicating gaps in treatment of 4 or more days, and collection of the proportion of days covered by medication dispensed. Finally, prevention behavior is defined as the number of unprotected sex acts by HIV partner status. All outcomes will be measured using both clinical record extraction and self-report; these two means of data collection will be compared for accuracy (using clinical record extraction as the gold standard).

**Table 1:** Measures, data sources, and data collection schedules for protocol exposures and outcomes

Domain	Instrument/measure	Data source	Data collection schedule
PRIMARY EXPOSURE			
Clinic randomization assignment	Clinic where participant receives care	Intake form	Baseline
SECONDARY EXPOSURE			
Intervention Dosage	Minutes of intervention received	PN participant contact forms and CommConnect system records	Ongoing capture
PRIMARY OUTCOMES			
Retention in HIV care	Patients on or initiating ART who remain on treatment 6 and 12 months later (with documented receipt of treatment)	Intake and Record extraction	Weekly (ART)
		Survey data (self-report)	0, 6, and 12 months
	Patients not ART eligible who have a repeat CD4 test at 6 months and 12 months after initial CD4	Intake and Record extraction	Bi-weekly (pre-ART)
		Survey data (self-report)	0, 6, and 12 months
SECONDARY OUTCOMES			
Linkage to HIV care	CD4 drawn and results available at clinic within 3 months of testing HIV positive	Intake and Record extraction	Weekly (ART) bi-weekly (pre-ART)
	CD4 result received within 3 months of testing HIV positive	Survey data (self-report)	0 and 6 months
	Patients eligible for ART who initiate treatment within 3 months of CD4 staging	Intake and Record extraction	Weekly (ART)
		Survey data (self-report)	0, 6, and 12 months
Adherence to ART	Proportion taking 90 % and 100 % of pills in the past 30 days	Survey data (self-report)	0, 6, and 12 months
	Breaks in treatment during the previous six months (at least 4 days in a row)	Intake and Record extraction	Weekly (ART)
		Survey data (self-report)	0, 6, and 12 months
	Proportion of days covered (# of dispensed days of medication/# of days between refills)	Intake and Record extraction	Weekly (ART)
Prevention (transmission risk)	Number of unprotected sex acts by partner HIV status	Survey data (self-report)	0, 6, and 12 months

Because the volume and intensity of the intervention might vary, particularly in the PN arm, we will also capture intervention exposure or dosage as a secondary exposure measure. Dose (minutes) of intervention received will be used for per protocol analyses. In the PN arm, this will include recording client data on frequency and duration of in-person meetings and phone communications with the navigator. Delivery and response to SMS messages for both intervention arms will be recorded with a standard duration of exposure (for example, 30 seconds) for each message, including two-way messaging. Any phone or in-person follow-up to SMS messages will be recorded separately. Secondary outcomes include linkage to HIV care, adherence to medications, and transmission risk behavior, as described in Table 1.

### Analysis

Our preliminary analysis will include presentation of participant demographic characteristics, service usage, acceptability of intervention approaches and self-reported sexual behavior within each of the three study arms using descriptive statistics (one-way and cross-tabular frequency tables and, for continuous variables, measures of central tendency). Measures of variability will account for repeated measures (multiple patients) and consequent correlation of variables (clustering) within each health facility. We will employ multiple imputation (MI) using chained equations to account for missing data. This procedure assumes that incomplete data arise from a missing-at-random (MAR) process, under which missingness is random conditional on observed covariables and observed outcomes rather than a missing-completely-at-random (MCAR) mechanism [43], thus making fewer assumptions about the nature of the missing data. We will conduct sensitivity analysis using weighted MI to evaluate the robustness of the MAR assumption [44].

Our primary analyses will employ an intention-to-treat (ITT) approach. We will employ logistic generalized estimating equations (GEE) to compare patient retention between intervention arms. We will also evaluate secondary outcomes (linkage, adherence and prevention behaviors) using an ITT analysis, as described above. We will employ GEE rather than mixed effects models as this approach allows us to estimate the effect of the intervention at the level of the population, rather than at the level of the individual. As our interventions will be implemented at the level of the health system, it is important to estimate the effect of the intervention at the level it is implemented [45]. Further, previous research has suggested that adjustment for confounding can improve the efficiency of estimates in the context of randomized trials [46]. We will thus conduct sensitivity analyses employing causal inference methods to account for any imbalance in distribution of site- and individual-level co-variables across study clinics. We will employ targeted maximum likelihood estimation [47], which allows estimation of both site- and individual-level co-variables, to minimize bias and improve efficiency of estimates of effect in the context of small sample sizes [48].

Our secondary analyses will employ a per-protocol approach to evaluate differences in primary and secondary outcomes. These analyses will resemble the intent-to-treat analyses described above, with the difference that individuals will be evaluated based on the number of minutes of intervention exposure they receive. As above, these analyses will account for the clustering of results within sites and include use of causal inference methods to adjust for potential confounding. These analyses will allow us to determine whether an observed effect of the intervention is limited due to inadequate implementation and whether there is a needed dosage for the interventions to successfully impact outcomes. This will also permit any accounting for contamination should an SOC participant also receive services at an intervention clinic (their intervention dosage can be included), though we do not believe this will be common based on distance between study clinics and experiences during the pilot study. Our primary and secondary analyses will employ robust standard errors as these are unbiased in a setting where the coefficient of variation is less than 0.20 [49]. Our sensitivity analyses will include computation of confidence intervals using bias-corrected sandwich estimators [49–51]. Stata, version 13, will be used to manage data and conduct the primary and secondary analyses; more complex sensitivity analyses will be conducted using R.

Finally, for the cost analysis, we will use the observed primary study outcome, retention in care to estimate the cost per person retained in each intervention arm. Costs will be summarized as total costs, cost per clinic and cost per individual enrolled in each intervention arm. Cost will also be broken out by pre- and post-implementation periods in order to assess incremental costs associated with intervention scale-up (each additional clinic added or individual enrolled). Efficiency will be measured as the cost per additional person retained in care for each arm of the study.

### Sample size estimation

We aim to enroll 750 patients across the 18 participating clinics, with the goal of ensuring that each of the 18 clinics has at least 30 participants, and no site more than 55 to guarantee a well-balanced sample and minimize variability. We anticipate that approximately 20 % of the sample will discontinue study participation, largely due to extensive migration in the area. This estimate of attrition is grounded in on our pilot experience, during which 10 % of participants moved out of the area during a 3-month observation period. We therefore anticipate an evaluable sample of 600 participants over 1 year. Under the assumption that only 50 % of participants in the SOC clinics will remain in care [10, 38], this sample size will provide 80 % power to observe a 20 % to 23 % difference in patient retention at 12 months between the PN and SOC or SMS and SOC arms and a 17 % to 20 % difference between the PN and SMS arms. These estimates assume a two-sided alpha of 0.05 and a coefficient of variation of 0.15 to 0.20 or an intra-cluster correlation of 0.0225 to 0.04 based on a review of the recent literature around sample size estimation in large cluster randomized trials [52, 53].

## Discussion

This trial will determine whether the use of SMS and peer-navigation interventions in rural and peri-urban clinics can improve engagement in HIV care and prevention in South Africa, where scalable interventions are sorely needed to improve current HIV outcomes. Both interventions being explored were pilot tested and demonstrated to be feasible to implement in similar low resource clinics in one of the study sub-districts (Moses Kotane) prior to beginning the trial.

The SMS approach is now being used broadly for health promotion given the ubiquitous presence of mobile phones in South Africa and other sub-Saharan African countries. Strategies utilizing cellular phones and other mobile technologies to improve HIV outcomes in sub-Saharan Africa are growing in number, particularly for adherence reminders [21, 54]. Additionally, the South African Government recently launched “MomConnect”, a national initiative for pregnant women that aims to improve access to maternal child health services by providing stage-based appointment reminders and well-baby information SMS messaging [55], demonstrating both national interest and support for this approach to engagement in care. The use of mHealth, particularly text messaging technologies, does bring with it potential challenges. In the pilot phase, participants were hesitant to reply to messages from our service for fear of airtime charge, despite assurances that all messaging to the project was free. A number of participants reported either losing their phone or having a break in coverage, necessitating constant phone number confirmation and updating. Finally, extensive efforts were needed to include all local carriers on the messaging service free of charge to the users, which required additional system programming. In summary, whereas the promise of mobile phones for improving HIV-related care outcomes is great, the need exists for additional research findings – showing not just that reminders can improve retention in care, but that the improved visit adherence will result in improved health [23].

The peer-navigation approach has also gained traction in recent years [21]. Though a peer-navigation intervention equivalent to this protocol has not yet been explored in South Africa to our knowledge, other treatment supporter interventions have demonstrated potential, pointing to the need for support that goes beyond mediation assistance to include emotional support and encouragement [56]. A community-based PN model may also be adapted to fit into the current policy climate in South Africa. The government is currently rolling out a new primary healthcare (PHC) re-engineering model, which includes the deployment of a cadre of community health workers to homes in their communities to monitor health. These ward-based outreach teams (WBOTs) are integrated into primary care facilities and support priority clinic services in the community, including HIV testing and HIV/TB care. Our team is exploring the possibility of integrating aspects of the peer navigation strategy within the WBOT model, pending trial findings. Another integration challenge that merits exploration in optimizing any community-based health model is how to incorporate the community-based work with services located at the clinic. We found in our pilot phase that integrating the peer navigators with clinical nursing and counseling staff was not easy, despite the fact that the clinic staff was pleased to have navigators working with the clinic patients. Other challenges include intensive training and supervision required to deploy and monitor navigators, who need ongoing mentoring and debriefing, particularly as they begin to traverse difficult barriers to care with their clients, such as alcohol dependence or food insecurity.

Although this trial will yield important data to drive programming in South Africa and surrounding countries, some limitations exist to potential inferences and the generalizability of the findings. Clinics selected for the study come from both peri-urban and rural areas; however, the selection area was limited to two sub-districts and included only those clinics with sufficient patient load to warrant inclusion. As a result, findings may not be generalizable to clients attending private clinics, to residents of other provinces, or to residents of large metropolitan centers or very rural areas where clinics have limited HIV patient load. Additionally, while trial participants are being systematically invited to participate and should represent the current patients undergoing HIV testing and receiving services at the clinics, the study population is not representative of the large number of residents who do not present for testing, which comprise approximately 35 % of the national adult population [57].

Results will provide crucial information regarding the efficacy of two complementary intervention strategies, with the recognition that one intervention (SMS) is less resource intensive but also less flexible in terms of the patient barriers to care that it can address. This trial is powered to compare retention outcomes at 12 months between the SOC and both the SMS and the PN intervention. This is an opportunity to estimate not only intervention efficacy but also incremental efficacy from the base SMS model to an enhanced peer navigation model with patient reminders (which could be generated either from an automated SMS system or directly from a peer navigator). In the future, an exploration of targeted interventions, or whether a more intensive intervention (PN) could be targeted to the clients with more personal barriers who require additional support, is needed. In addition, knowledge of whether the less intensive model of SMS would be sufficient for those who need reminders but do not experience major barriers, such as fear of disclosure or substance use, is also needed.

## Trial status

The trial began recruitment in one of two study sub-districts on 20 October 2014 and in the second sub-district on 26 January 2015. We aim to complete recruitment by 5 January 2015.

## Abbreviations

ART: antiretroviral therapy
DoH: Department of Health
GEE: generalized estimating equations
HIV: human immunodeficiency virus
ITT: intent to treat
PHC: primary health clinic
PN: peer navigation
SMS: short messaging service
SOC: standard of care
